# Knowledge, Attitudes, and Practices of Artisanal and Small-Scale Miners regarding Tuberculosis, Human Immunodeficiency Virus, and Silicosis in Zimbabwe

**DOI:** 10.3390/ijerph20237116

**Published:** 2023-11-27

**Authors:** Dingani Moyo, Fungai Kavenga, Ronald Thulani Ncube, Farai Peter Macheri, Tariro Christwish Mando, Florence Moyo, Orippa Muzvidziwa, Mpokiseng Ncube, Hellen Masvingo, Blessings Chigaraza, Andrew Nyambo, Albert Mangwanya, Rosemary Ncube Mwale, Trust Mazadza, Tinashe Magidi, Gerald Benny, Chamunorwa Ndudzo, Victoria Varaidzo Kandido, Kudzaishe Mutungamiri, Collins Timire

**Affiliations:** 1 Baines Occupational Health Services, Harare P.O. Box 1008, Zimbabweorippam@bainesohs.org (O.M.); mpokisengn@bainesohs.org (M.N.); hellenm@bainesohs.org (H.M.); blessingsc@bainesohs.org (B.C.); 2 Faculty of Medicine, National University of Science and Technology, Bulawayo P.O. Box AC 939, Zimbabwe; 3 School of Public Health, University of the Witwatersrand, Johannesburg 2017, South Africa; 4 Ministry of Health and Childcare, Harare P.O. Box CY 1122, Zimbabwe; drkav8@gmail.com (F.K.); macherif@staff.msu.ac.zw (F.P.M.); tariro.christwish@gmail.com (T.C.M.); andrewnyambo@gmail.com (A.N.); amangwanya9@gmail.com (A.M.); rosemarymwale@gmail.com (R.N.M.); tmazadza18@gmail.com (T.M.); tinashemagidi03@gmail.com (T.M.); bennygerald@gmail.com (G.B.); chamunorwandudzo@gmail.com (C.N.); collinstimire2005@yahoo.com (C.T.); 5 Union Zimbabwe Trust, Harare, Zimbabwe; rncube@uzt.org.zw (R.T.N.); vchizana@uzt.org.zw (V.V.K.); 6 Department of Internal Medicine, Faculty of Medicine, Midlands State University, Gweru P.O. Box 9055, Zimbabwe; 7 Department of Health Sciences, Faculty of Sciences, Zimbabwe Open University, Harare P.O. Box 1119, Zimbabwe; 8 Jointed Hands Welfare Organization, Gweru P.O. Box 1945, Zimbabwe; kmutungamiri@jointedhands.org

**Keywords:** occupational exposure, mineworker, Zimbabwe, silico-tuberculosis

## Abstract

In Zimbabwe, artisanal and small-scale miners (ASMs) have a high prevalence of tuberculosis (TB), human immunodeficiency virus (HIV), and silicosis. Previous studies on ASMs utilised programme data, and it was not possible to understand reasons for the high prevalence of these comorbidities. We conducted a cross-sectional study to investigate the knowledge, attitudes, and practices of ASMs regarding TB, HIV, and silicosis. We enrolled a convenience sample of 652 ASMs. Their mean (standard deviation) age was 34.2 (10.8) years. There were 602 (92%) men and over 75% had attained secondary education. A total of 504 (80%) of the ASMs knew that TB is a curable disease, and 564 (87%) knew that they were at higher risk of TB than the general population. However, they were less likely to know that HIV increases the risk of TB disease, 340 (52%), with only 226 (35%) who perceived the risk of TB infection to be high among ASMs. Only 564 (59%) were aware that silica dust causes permanent and incurable lung diseases. Six hundred and twenty (97%) showed a positive attitude towards healthcare when they were sick, and 97% were willing to use special respirators to prevent dust inhalation. On practices, only 159 (30%) reported consistent use of either cloth or respirators to prevent dust inhalation. Three hundred and five (49%) ASMs reported consistent use of condoms outside their homes and 323 (50%) reported use of water to suppress dust. Only 480 (75%) of ASMs sought healthcare services when sick. ASMs cited challenges of accessing healthcare services due to lack of money to pay for healthcare (50%), long distances to clinics (17%), and the shortage of medicines at clinics (11%). Effective control of TB, silicosis, and HIV among ASMs requires addressing the identified knowledge gaps and barriers that are faced by ASMs in accessing personal protective equipment and healthcare services. This will require multisector collaboration and the involvement of ASMs in co-designing a package of healthcare services that are tailored for them.

## 1. Introduction

An estimated 40 million people are directly engaged in artisanal and small-scale mining, with an additional 150 million people dependent on this sector across at least 80 countries [1,2,3,4]. In Africa, there are around 10 million artisanal and small-scale miners (ASMs), and around 54 million people are reliant on them for their livelihoods [1,2,5]. Among ASMs in Africa, 40–50% are women, and 80% are classified as part of the informal sector [1,2,6].

In Zimbabwe, the estimated number of ASMs is around 500,000 with two million people dependent on them, although this number is probably much higher [7]. ASMs contribute an average of 60–77 percent of the gold production in Zimbabwe [8,9].

Artisanal and small-scale mining is generally poverty-driven and labour-intensive work, performed using archaic mining methods [10,11,12]. It is associated with multiple health and safety hazards that leave miners vulnerable to occupational health risks and diseases. Common morbidities include TB, silicosis, human immunodeficiency virus/acquired immunodeficiency syndrome (HIV/AIDS), sexually transmitted infections (STIs), malaria, malnutrition, and mercury neurotoxicity [13]. Miners are also at risk of multiple mental health issues, such as anxiety, depression, fatigue, and substance use [14,15,16]. Silicosis is a progressive disease with more than 30% of the cases progressing despite removal from exposure to silica dust [17]. Improving mining practices needs education and awareness around the attendant occupational hazards.

The prevalence of TB, HIV, and silicosis is high among ASMs. A recent study showed the prevalence of silicosis at 19%, that of TB at 6766 per 100,000, and HIV at 18%, much higher than the national prevalence of 240 per 100,000 and 12.9%, respectively [18,19]. What is particularly concerning is the high proportion of young people who are affected [18]. Silicosis or silica dust exposure poses a lifetime three- to four-fold increased risk of TB infection, while the risk of TB in patients with HIV and silicosis is in excess of 15-fold [19,20,21]. Co-morbidities (silico-TB, TB/HIV, etc.) can lead to poor treatment outcomes.

Regular screening can help detect silicosis, TB, and HIV early so that ASMs can be included in medical care. Given that ASMs are highly mobile and operate in remote areas, often with poor access to health services, interventions that improve access to screening services are crucial. In Zimbabwe, the Zimbabwe Miners Federation (ZMF) has made efforts to regularize artisanal and small-scale mining (about 40,000 of at least 500,000 are reported to have registered) [22]. In addition, the Kunda Nqob’i TB (KNTB) project in partnership with the Ministry of Health and Child Care has rolled out interventions such as targeted screening for active TB, occupational health clinics, workplace-based screenings (WBS), and occupational health camps to screen, diagnose, and link ASMs to TB, HIV, and silicosis care. The prevalence of these conditions has been documented to be high in such settings [23,24]. In view of the huge burden of disease, it is critical to understand the knowledge, attitudes, and practices of ASMs with regards to TB, HIV, and silicosis to inform targeted interventions to address this triple burden among the artisanal and small-scale mining population. We, therefore, set out to evaluate the knowledge, attitudes, and practices of ASMs regarding TB, HIV, and silicosis.

## 2. Methods

### 2.1. Study Design

This was a descriptive cross-sectional study on ASMs attending workplace-based screenings (WBS) for TB, HIV, and silicosis at two occupational health clinics and mobile workplace-based facilities in the Midlands and Matabeleland South provinces.

### 2.2. General Setting

Zimbabwe is a landlocked country in southern Africa with a population of 15.1 million spread across 10 provinces and 91 administrative districts [25]. It is bordered by Mozambique in the east, South Africa in the south, Botswana in the west, and Zambia in the north. Mining is one of the major economic activities in the country, contributing almost 12% of GDP [2]. Most mining activities, both large-scale and artisanal and small-scale mining, occur along the Great Dyke, approximately 550 km long and 3–12 km wide. It is rich in gold, diamonds, platinum, chrome, asbestos, and tin [3]. Zimbabwe’s TB incidence rate declined from 242 per 100,000 in 2015 to 190 per 100,000 in 2021 [4]. The HIV prevalence is 12.9% [18], with the highest in Matabeleland South (17.6%).

### 2.3. Specific Setting

The study was carried out in the Midlands and Matabeleland South provinces. There are eight districts implementing the KNTB project funded by the United States Agency for International Development (USAID). Of the eight districts, Gwanda (Matabeleland South province), Gweru, and Chirumhanzu (Midlands province) were conveniently selected since data collection for the study coincided with TB and silicosis screening activities in these districts. Artisanal and small-scale mining activities include alluvial, surface, and underground mining for gold, which is carried out using rudimentary mining methods. However, chrome is also mined using both open-cast and underground mining methods. Most men are involved in underground mining where the ventilation is poor, whilst women mostly do surface mining.

The districts, particularly Gwanda, are in low rainfall regions associated with huge challenges in access to water [26]. In these districts, ASMs usually operate in remote areas, making access to water, healthcare, and other social services a greater challenge. Even where water is available, lack of mechanization hinders the accessibility of the resources at the mines. Apart from the mining activities of ASMs in these areas, women are involved in the trade of various wares. A lot of small-scale businesses have sprouted, including food outlets and mini-bars [27]. The bars are night drinking spots and are frequented by female sex workers [28,29]. Usually, ASMs finish work in the evenings and attend food outlets and bars.

### 2.4. Screening for TB/HIV and Silicosis

As part of the KNTB project, all ASMs are screened for TB, HIV, and silicosis free of charge. Screening is either workplace-based (outreach screening undertaken at artisanal and small-scale mining sites) or at two occupational health clinics (OHCs) set up within the premises of the Gwanda and Gweru provincial hospitals, both of which are public health institutions. ASMs are either self-referred or referred by surrounding health facilities or following workplace-based screening.

### 2.5. Mobilization of ASMs for WBS

About one or two weeks prior to WBS, a two-day mobilization exercise is carried out in surrounding artisanal and small-scale mining areas. This mobilization exercise is conducted by representatives from the district health offices, the ASM local leadership, the Baines Occupational Health Services (BOHS), and other partners implementing other projects within the artisanal and small-scale mining sites. The purpose of the two-day mobilization visits is to raise awareness about the forthcoming WBS and to identify worksites with high numbers of ASMs where the four-day screening visits would be held.

### 2.6. Study Population

The study population consisted of adult men and women who identified themselves as ASMs and were confirmed as such by the attending nurses at the health facilities through occupational history taking. These ASMs presented to the OHCs and mobile workplace-based health facility in Gweru, Chirumhanzu, and Gwanda districts from 16 May 2023 to 31 July 2023.

### 2.7. Sampling Procedure

The minimum sample size was calculated using the Dobson formula, with the following assumptions: a prevalence of 50%, and a precision of 0.05 at the 95% confidence interval, with an expected minimum sample size of 384. After factoring a non-response rate of 10%, the final sample size was 423.

Convenience sampling was carried out. All ASMs presenting for screening during WBS and at the two OHCs were eligible for inclusion in the study. Firstly, the ASMs were briefly informed about the study, including the purpose, the duration, as well as the risks and benefits. They were additionally given a participant information sheet to read about the study, and, for those who could not read, the research assistants read the information sheet for them in their preferred language. ASMs who expressed interest were enrolled in the study, after providing a written informed consent. ASMs who were too sick to participate in the study were excluded. Questionnaires were interviewer administered across the three main local languages, namely English, Ndebele, and Shona.

### 2.8. Data Variables, Sources of Data, and Data Collection

The following data variables were collected as part of this study: sociodemographic data (sex, date of birth, date of interview, duration as a miner, level of education, and district); knowledge (whether TB is curable; silicosis is transmissible from one person to the other; a piece of cloth can prevent dust inhalation); practices (what ASMs use to prevent dust inhalation; health-seeking behaviour for medical care whenever one gets sick; frequency of use of respirators; condom use during sex outside home), and challenges in accessing medical care. Individual level data were collected using a structured questionnaire during the study period from 16 May to 31 July 2023.

### 2.9. Data Analysis

Individual level data were entered in EpiData v 3.1 (EpiData Association, Odense, Denmark) and were analysed using EpiData Analysis v 2.2.3.187 and Stata v 13.0 (StataCorp, College Station, TX, USA). Categorical variables were summarized using frequencies and proportions. Age was calculated as the difference between the date of interview and the date of birth to obtain the age in days. The days were divided by 365 to obtain the age in years. Initially, continuous variables, such as age and duration as a miner, were assessed for normality by plotting histograms and using the Shapiro–Wilk test. Age was summarized using means and standard deviations, and experience as a miner was summarized using medians and the interquartile range.

### 2.10. Ethical Approval

Permission to conduct the study was obtained from the Permanent Secretary for Health, the Ministry of Health and Child Care (MoHCC), and from the artisanal and small-scale association national leadership. Ethics approval was obtained from the Medical Research Council of Zimbabwe (MRCZ), approval number (MRCZ/A/3022). All study participants gave written informed consent to take part in the study.

## 3. Results

A total of 670 ASMs were reached during WBS and the two OHCs. Of these, 652 (97%) consented to take part in the study. Their socio-demographic characteristics are shown in Table 1. More than 90% were men and more than 75% had attained at least secondary level education. The mean age was 34.2 and the standard deviation (SD) was 10.8 years. More than 60% were in the age categories 25–44 years. Most ASMs were enrolled from Gwanda (55%), followed by Chirumhanzu (36%) and Gweru (9%). Of the 609 ASMs who had documented years of experience as a miner, their median years as miners was 5 years, interquartile range (IQR) (3–10 years). The majority (57%) of ASMs had mining experience of at least 5 years, the maximum being 32 years.

## 4. Knowledge, Attitudes, and Practices Regarding HIV, TB, and Silicosis among ASMs

### 4.1. Knowledge about HIV, TB, and Silicosis

Artisanal and small-scale miners were knowledgeable, 504 (80%), that TB is a curable disease and that they had a higher risk of TB compared to the general population, 564 (87%). However, they were less likely to know that HIV increases the risk of TB disease, 340 (52%), with only 226 (35%) who perceived the risk of TB infection to be high among ASMs, as shown in Table 2.

Conversely, only 564 (59%) had correct knowledge that silica dust causes permanent and incurable lung disease and only 192 (30%) had accurate knowledge that silicosis is not transmitted from one person to the other. Of note, more than 90% reported the importance of yearly tests for TB, HIV, and silicosis among ASMs, and the use of special respirators to prevent dust inhalation.

### 4.2. Attitudes and Practices among ASMs regarding HIV, TB, and Silicosis

Artisanal and small-scale miners had positive attitudes about seeking healthcare services whenever they are unwell and accessing TB, HIV, silicosis screening and testing services (97%), and using special respirators to prevent dust inhalation (90%), Table 3.

However, positive intentions by ASMs to reduce dust inhalation, seek medical care, and to prevent HIV infection do not translate into practices, as shown in Table 4. The most common method of preventing dust inhalation was the use of a piece of cloth, reported by 447 (69%). Of the 529 ASMs who reported use of either a piece of cloth or special respirators, only 159 (30%) reported consistent use every time. Half of ASMs never used water to suppress dust during mining activities and just under 50% reported using condoms every time they have sex outside their homes. In terms of health seeking, almost three-quarters of ASMs reported they seek healthcare whenever they feel unwell.

### 4.3. Key Challenges in Accessing Medical Care

**Figure 1:**
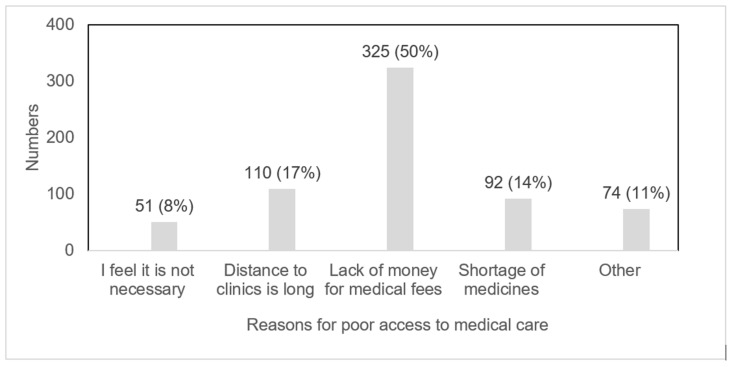
Reasons for poor access to medical care among study participants.

Of the 652 ASMs who responded to the question on key challenges in accessing medical care, almost 50% cited lack of money to pay for medical fees, followed by barriers such as long distances to clinics, Figure 1.

### 4.4. Possibilities of Reducing TB, HIV, and Silicosis among ASMs

Of the 649 ASMs who responded to the question whether it is possible to reduce TB, HIV, and silicosis in mining, 473 (73%) responded positively. The 68 (11%) who responded negatively cited reasons that ranged from lack of condoms, lack of self-control over sexual desires among ASMs, to overcrowded living conditions and the high exposure of ASMs to women. Some of the reasons increased the chances of unprotected sex.

## 5. Discussion

We found a predominance of men in artisanal and small-scale mining. There were higher knowledge levels about TB than silicosis. Most ASMs had poor knowledge on effective respiratory protective measures. Almost all ASMs expressed willingness to attend regular screenings for TB, HIV, and silicosis and to seek medical care whenever they became sick. However, access to medical care was reportedly constrained by barriers, such as long distances to healthcare facilities, lack of money for consultations, and stock-outs of medicines at clinics. ASMs engage in practices such as unprotected sex outside their homes which exposes them to HIV. They also do not adhere to measures that either suppress or prevent inhalation of dust which puts them at risk of silicosis.

The predominance of men among ASMs is an expected finding. Several studies have reported higher proportions of men compared to women in artisanal and small-scale mining [19,30,31,32]. Artisanal mining is a labour-intensive exercise and is usually carried out in remote areas that are characterised by health and safety risks, violence, and poor access to health and sanitary facilities [19,31,33,34,35]. These could be push factors for women, especially in our setting.

Tuberculosis and HIV are relatively well-known conditions, and health promotion messaging in both health facilities and on media is usually undertaken to raise awareness about these conditions. Given that the prevalence of HIV is high among ASMs in Zimbabwe (19), it is possible that a greater number of ASMs may have had previous contacts with health facilities and received health information regarding TB and how HIV increases the chances of developing TB. Zimbabwe has strengthened TB/HIV collaborations and people with TB are routinely screened for HIV and vice-versa as per the national TB and HIV management guidelines [36,37]. The high knowledge levels observed in our study are, thus, both expected and consistent with results from Zambia [38]. However, ASMs lacked knowledge that HIV increases the risk of TB.

In our study, there were low knowledge levels about silicosis, with prevalent unsafe practices, such as the use of cloth to prevent dust inhalation and inconsistent or absent dust suppression measures. In other settings, knowledge levels about silicosis were also reported to be low [39,40]. This is not surprising as silicosis is a relatively unknown condition and is almost absent in most health promotion messaging. It is known that both the duration and intensity of exposure to silica dust increase the risk of TB and silicosis [20,33,41,42]. A high prevalence of silicosis is expected among miners with many years of mining experience. Previous studies on ASMs in Zimbabwe revealed a high prevalence of silicosis of up to 19%, despite a relatively young population who had few years of mining experience [19]. This study confirms the findings from previous studies on unsafe mining practices, such as the use of cloth and lack of, or inconsistent, dust suppression measures during mining activities by ASMs [19,30]. In addition, the perception of drinking milk or juice to clear dust in the lungs is a misconception that may also explain the inconsistent use of dust inhalation preventive measures. The high prevalence of silicosis in young ASMs in Zimbabwe is probably due to a high intensity of exposure to silica-containing dust during the few years they are involved in mining activities. It should be noted that ASM activities are driven by poverty, and some ASMs may either lack knowledge about prevention methods or are constrained by resources to adhere to such. Rudimentary mining methods increase exposure to silica dust. This is compounded by the scarcity of water and equipment to suppress dust during mining operations.

For a long time, artisanal miners have been blamed for their seemingly poor health-seeking behaviour [12,43]. Contrary to this view, a growing body of evidence from southern Africa shows that this is not the case [38,44,45]. Our findings are consistent with this growing body of evidence as we observed that almost all ASMs are keen on having regular screenings for HIV, TB, and silicosis, while those who get sick are willing to seek medical care. However, ASMs usually face barriers to translate these positive intentions into good health-seeking behaviour and practices. These barriers range from structural ones related to long distances to clinics to lack of money to pay for consultations and the shortage of medicines in health facilities. Usually, ASMs operate in remote and hard-to-reach areas, making contact with health facilities a challenge. Other reasons for delayed health-seeking as observed in this study, included rigid clinic operating hours, long waiting times at health facilities, and the fear of leaving mining activities and losing their share of gold/money while they are away attending to their healthcare needs.

There are quite a number of health-related risks within the environment when ASMs carry out their activities. These include communicable diseases, such as HIV and other sexually transmitted infections (STIs). Several factors that fuel the spread of HIV, including STIs, were reported by ASMs. These include a lack of condoms, including their inconsistent use in sexual relations outside their homes. ASMs operate in remote places far away from their hometowns. Female sex workers frequent shops and bars that are located in mining areas. This may fuel multiple concurrent and sexual relationships and the transmission of STIs and HIV.

Our study has two key strengths. Firstly, the data collection was carried out at mining sites after a mobilization exercise by a team of people drawn from representatives of ASMs. This ensured a fairly good sample size and a high response rate. Secondly, all people who availed themselves were eligible for the study.

However, this study has some limitations. By restricting enrolment to the three districts of Chirumhanzu, Gweru, and Gwanda, our results may not be generalizable to the whole population of ASMs in Zimbabwe. Furthermore, we did not capture data on TB, HIV, and silicosis concurrently with data collected on knowledge, attitudes, and practices. This was a missed opportunity to establish associations between particular behavioural practices and outcomes, such as TB, HIV, and silicosis. Finally, we cannot rule out the “healthy worker effect” [46]. People who died or were too sick to avail themselves at work-based screening sites and at the two occupational health clinics may have had worse knowledge and practices and, thus, were more likely to be sicker than those who attended WBS and were enrolled in the study. However, since screening services were brought to the sites of operation by ASMs, even sicker ASMs may have seen it as an opportunity to be screened and treated for ailments compared to healthier ASMs who may not have had any incentive to attend the screening services.

Despite these limitations, our results have some implications for both policy and practice. Firstly, poor health-seeking behaviour among ASM is a myth. Instead, ASMs are constrained by several barriers to accessing health services. There is a need to ensure participation of ASMs in co-designing the type and mix of health services that are tailored to their needs [38]. This may include clinic operating hours that are flexible to ASM schedules, friendly staff, and better clinic scheduling to minimize waiting times. On the health front, there is a need to ensure that clinics are adequately stocked with critical medicines.

Artisanal and small-scale miners usually operate in hard-to-reach areas which may be far away from static clinics. It is important to bring health services to them, since static clinics may not serve the purpose of this particular group. Perhaps this explains the success of the KNTB mobile WBS. Services provided will need to be expanded to include services for STIs to ensure holistic and patient-centred care. However, considerations need to be made to reducing stigma, which is usually associated with the provision of TB, STI, and HIV services. To mitigate this, the scope of services may be expanded to include free screenings for non-stigmatized conditions, such as blood pressure, blood sugar, nutritional status, and the provision of contraceptives and condoms.

There is also need for health promotion messaging addressing the information gaps identified. There were mixed understandings of preventive efforts but an openness to engage with healthcare services.

## 6. Conclusions

The effective control of TB, silicosis, and HIV among ASMs requires addressing the identified knowledge gaps, provision of personal protective equipment, and engaging ASMs to co-design a package of healthcare services that are tailored for them. This will require collaboration among the Ministry of Health, artisanal and small-scale miners’ associations of Zimbabwe, the Zimbabwe Miners Federation, and representatives of ASMs.

## Figures and Tables

**Table 1:** Sociodemographic characteristics of ASMs who were enrolled in the study in Chirumhanzu, Gweru, and Gwanda districts, 2023, *n* = 652.

Characteristic		Number ((%) ^§^)
Sex	Male	602 (92)
	Female	43 (7)
	Not recorded	7 (1)
Age category in years	<20	34 (5)
	20–24	102 (16)
	25–34	236 (36)
	35–44	167 (26)
	≥45	101 (15)
	Not recorded	12 (2)
Level of education	None	37 (6)
	Primary	104 (16)
	Secondary	454 (70)
	Tertiary	45 (7)
	Not recorded	12 (1)
District of operation	Gwanda	362 (56)
	Chirumhanzu	236 (36)
	Gweru	54 (8)
Years as a miner (*n* = 609)	<5	262 (43)
	5–9.9	169 (28)
	≥10	178 (29)

§ = Column percentages.

**Table 2:** Knowledge about TB, HIV, and silicosis among ASMs who were enrolled in the study in Chirumhanzu, Gweru, and Gwanda districts, 2023.

Characteristic	No (%) ⁑	Yes (%) ⁑	I Don’t Know (%) ⁑
TB can be cured (*n* = 633)	18 (3)	504 (80)	111 (17)
Dust generated during mining can cause TB	30 (5)	571 (88)	50 (8)
HIV increase chances of contracting TB	73 (11)	340 (52)	236 (36)
ASM have higher risk of getting TB compared to general population	40 (6)	564 (87)	48 (7)
Silica dust causes permanent and incurable lung disease (*n* = 650)	81 (13)	382 (59)	187 (29)
Silicosis can be transmitted from one person to the other (*n* = 648)	192 (30)	224 (35)	232 (36)
The risk of getting TB is low in ASMs	332 (51)	226 (35)	89 (14)
Use of a piece of cloth to cover one’s mouth and nose is a good way to prevent dust inhalation.	189 (29)	434 (67)	28 (4)
Drinking milk or juice clears dust in the lungs and prevents TB.	135 (21)	434 (67)	82 (13)

ASMs = Artisanal and small-scale miners; OLD = Occupational lung disease; ⁑ = Row percentages.

**Table 3:** Attitudes about TB, HIV, and silicosis among ASMs who were enrolled in the study in Chirumhanzu, Gweru, and Gwanda districts, 2023.

Characteristic	No (%) ⁑	Yes (%) ⁑	I Don’t Know (%)
It is crucial for ASMs to have yearly tests for TB, HIV, OLDs	6 (1)	625 (96)	21 (3)
It is necessary to buy special respirators to prevent dust inhalation	29 (4)	595 (91)	28 (4)
Wearing a respirator, not a piece of cloth during mining, is good.	28 (4)	574 (88)	48 (8)
It is important for ASMs to always have access to condoms.	50 (8)	584 (90)	16 (2)
It is possible to reduce HIV, TB, and silicosis in mining.	68 (11)	475 (73)	106 (16)
It is important to always seek medical treatment when one is not feeling well.	15 (2)	620 (97)	7 (1)
I would participate if TB, HIV, and silicosis screening services were brought to mining sites.	14 (2)	620 (97)	6 (1)

ASMs = Artisanal and small-scale miners; OLD = Occupational lung disease; ⁑ = Row percentages.

**Table 4:** Practices among ASMs who were enrolled in the study in Chirumhanzu, Gweru, and Gwanda districts, 2023, *n* = 652.

Characteristic		Number (%) ^§^
Method for preventing dust inhalation (*n* = 646)	Nothing	117 (18)
	A piece of cloth	447 (69)
	Special respirators	82 (13)
Frequency of use of cloth or respirator (*n* = 532)	Rarely	53 (10)
	Sometimes	231 (43)
	Most of the times	89 (17)
	Every time	159 (30)
Use water to suppress dust (*n* = 645)	Never	322 (50)
	Sometimes	186 (29)
	Most of the times	53 (8)
	Every time	84 (13)
Condom use during sex outside home (*n* = 628)	Never	85 (14)
	Sometimes	141 (23)
	Most of the times	97 (15)
	Every time	305 (49)
I seek medical care whenever I am sick (*n* = 649)	Yes	480 (74)
	No	169 (26)

§ = Column percentages.

## Data Availability

The data presented in this study are available on request from the corresponding author. The data are not publicly available due to authorisations that may be required by the Ministry of Health and Child Care of Zimbabwe.
